# Notes and News

**Published:** 1905-10-28

**Authors:** 


### NOTES AND NEWS.

The bazaar in aid of the West Ham Hospital which we announced for October 26 has been postponed to November 23, 24, and 25 in order to enable the President, the Duchess of Marlborough, to attend. Princess Louise of Schleswig Holstein will open the bazaar on the first, and the American Ambassador will do so on the second day.

The members of the Russian Red Cross Society who assisted in the war with Japan are now being dispersed. It is stated that 500 medical men and 4,000 Sisters of Mercy were enrolled for duty.

The Moseley water supply, near Manchester, is under suspicion, traces of lead have been found in a number of samples, and a good many cases of lead poisoning have been reported.

The German Association of Naturalists and Medical Men met this year at Meran. A considerable amount of attention was paid to the discussion of pellagra, which is especially prevalent in Austria and Hungary, and Professor Neusser proposed the creation of an international institute for the study of the disease.

On the 27th instant Sir Frederick Treves will give an address on "The Army Medical Service," at the Royal Pavilion, Brighton, on the occasion of the inaugural meeting of the Brighton and Sussex Medico-chirurgical Society. The Society are issuing invitations through Dr. Newsholme, their President.

It is mostly from some State or other in America that the most drastic legislation emanates. The evils attending on the marriage of the unfit have long been recognised, but the honour of establishing preventive measures appears likely to fall to Colorado. A Bill is now before the Legislature which requires all marriages to be sanctioned by the State, who will demand a physical examination before granting the necessary license.

Cholera in Germany seems to be subsiding, but precautions are not relaxed, as the disease is said to be still prevalent in Russia.

The Annual Festival of the Newsvendors' Benevolent and Provident Institution, at which Sir Horace Marshall is to preside, will be held on October 31 at De Keyser's Hotel, Blackfriars.

Monsieur Gerault-Richard, editor of the *Petite Republique*, will ask the French Parliament to vote £4,000 for research work, with the view to the discovery of a cure for tuberculosis.

The annual dinner of the Hospital Officers' Association was held on October 25 at the Gaiety Restaurant, the President of the Association, Mr. P. Michelli, being in the chair. Lord Sandhurst was the guest of the evening, and the large company present included representative members of the hospital world, both of London and the provinces.

The objections which arise when a female resident medical officer is appointed have been cited at the Aston Workhouse, where Dr. Catherine Fraser has been elected. A suggestion is made that a male locum tenens might be provided during the absence of the chief medical officer. No doubt this suggestion must be followed in cases where there is a difficulty in finding male candidates to fill the post, but otherwise the arrangement is liable to rejection on the score of expense.

It will be remembered that several persons in the crowd in Holborn on the occasion of the opening of Kingsway were knocked down by a carriage, in which were members of the Paris Municipal Council. Before leaving England, MM. Rebrillard and Henri Rouselle, Vice-Presidents of the Council, called at King's College Hospital to inquire after one of the injured persons who was conveyed thither. Later a present of £10 followed the visit, but unfortunately the patient had died from her injuries.

Twelve of the Middlesex Urban and Suburban District Councils agreed, late in the year 1903, to apply to the Local Government Board for a provisional order forming their districts into a Joint Small-pox Hospital District. In 1904 the necessary agreements to combine were executed, a Joint Hospital Committee was formed, and the Association called the Middlesex Joint Small-pox Board. The Board are of opinion that they can purchase and maintain a suitable hospital at a cost to the constituent Councils of not more than the sum produced by a rate of one halfpenny in the pound per annum. It is intended for the present to have one large hospital, with motor ambulances to carry patients to it from any of the combined districts, and the Joint Board believe they will be able to cope in an adequate manner with any outbreak of small-pox that may occur in the future. Under the joint scheme, all of the constituent Councils will contribute to the whole of the expenses, according to their rateable value.

